# Factors associated with high viral load among HIV clients aged 15 years and older receiving treatment in Tanga city council, Tanzania: A facility-based cross-sectional study

**DOI:** 10.1371/journal.pone.0348862

**Published:** 2026-05-07

**Authors:** Aqbara Ibrahim Chande, Novatus A. Tesha, Bruno Sunguya

**Affiliations:** Muhimbili University of Health and Allied Sciences, School of Public Health and Social Sciences 9, United Nations Road, Upanga West, Dar es Salaam, Tanzania; University of Zimbabwe Faculty of Medicine: University of Zimbabwe College of Health Sciences, ZIMBABWE

## Abstract

**Background:**

High viral load indicates poor treatment outcomes among people living with HIV (PLHIV) on antiretroviral therapy (ART). However, there is a dearth of evidence on specific factors associated with high viral load in resource-limited settings, including Tanzania.

**Aim:**

The aim of this study is to identify factors contributing to high viral load among PLHIV aged 15 years and older on ART for at least six months in Tanga, Tanzania.

**Methods:**

This is analytical cross-sectional study of 233 PLHIV attending the Care and Treatment Centre (CTC) in Tanga region from September to November 2023. A systematic sampling method was used to select participants for face-to-face interviews. A structured questionnaire was used to collect socio-demographic information while clinical data were collected from the patients’ records and CTC database. Descriptive analysis was used to estimate the prevalence of high viral load, while Pearson Chi-square tests compared categorical variables, and the logistic regression assessed determinants of high viral load.

**Results:**

High viral load was prevalent among 35.2% [95% CI: 29.3%−41.6%] PLHIV attending CTC in Tanga region. Higher viral load was noted among younger adults (52.5%), those in sales/services (63.6%), professionals (54.5%), and unskilled workers (53.2%) compared to their counterparts. PLHIV with severe food insecurity were more likely to exhibit higher viral load compared to those from food secure households (AOR = 9.6; 95% CI: 2.6–35.2). Those consuming alcohol were 4.2 times more likely to have a higher viral load compared to non-drinkers (AOR = 4.2; 95% CI: 1.4–12.5). PLHIV aged 20–24 were 5.4 times more likely to exhibit elevated viral load levels compared to their older counterparts (AOR = 5.4; 95% CI: 1.8–15.7). Additionally, PLHIV in sales and services occupations were 6.1 times more likely to have a higher viral load compared to those in agriculture (AOR = 6.1; 95% CI: 1.1–35 and those facing high stigma were 4.3 times more likely to have a higher HVL than individuals with good social support and low stigma (AOR = 4.3, 95% CI: 1.0–18.7, p = 0.049).).

**Conclusion:**

More than one in three adult PLHIV on ART in Tanga Tanzania had a high viral load. This burden highlights a steep climb to reach the last 95 target ahead of the deadline. Efforts should focus on young adults, those with households’ food insecurity, consuming alcohol, and with perceived stigma in Tanzania and areas with similar context.

## Background

To combat the burden of HIV, the joint United Nations Programme on HIV/AIDS(UNAIDS) launched the “95-95-95” targets in 2015 [1]. The third 95 focuses on viral suppression, where only 53% had reached the agreed target globally [2]. Addressing the non-viral suppression can mitigate the burden of advanced HIV disease, prolong the lives of People Living with HIV (PHLIV), and therefore HIV transmissibility. Advanced HIV disease defined as clinical stages 3 or 4 is marked by severe symptoms like opportunistic infections and significant weight loss, alongside a CD4 count below 200 cells/mm³ [3]. Viral Load Suppression (VLS) is a crucial marker of treatment efficacy in HIV-positive patients and is considered achieved when the reading is less than 1,000 HIV RNA copies/mL of plasma. Despite the effective implementation of *Antiretroviral Therapy (*ART) initiatives in countries with high burden [4], individuals suffering from advanced HIV disease continue to present to hospitals, and as a result, medical admissions and deaths due to HIV-related infectious diseases remain high.

In Tanzania, the adult HIV prevalence rate is 4.4%, with higher rates in women (5.6%) than in men(3.0%) [5]. VLS among adults aged 15 years and older in Mainland Tanzania is 78.1% [5]. According to Tanzania’s ART recommendations, successful ART reduces HIV Viral Load (HVL), promotes immunological recovery, and hence increases CD4 cell count [6]. The Tanzania HVL testing guideline, recommends measuring CD4 counts every six months to monitor the immune response to ART [6]. HVL testing is also utilized alongside clinical and immunologic data to diagnose treatment failures more promptly, despite its limited capacity and availability [6,7].

Tanzania remains a priority country for HIV and acquired immune deficiency syndrome (AIDS) in sub-Saharan Africa, with approximately 1.7 million PLHIV as of 2019 year [8]. Njombe region in the Southern highlands of Tanzania reported the highest prevalence at 11.4%, while Tanga in the coastal areas had a moderate rate of 5.0% [8], although higher than the national average. The country has adopted the updated UNAIDS fast track goals to end the AIDS pandemic by 2030, aiming for 95% of PLHIV to know their status, 95% on treatment, and 95% of those on ART to be virally suppressed [9]. By 2022, 83% of HIV-positive individuals were aware of their status, 95% were on ART, and 92% of those on therapy had achieved VLS [9]. These rates of reaching these targets are not similar in all sub-population in the country. For example, the prevalence of VLS among adults aged 15 and older in Mainland Tanzania is only 78.1% [10]. This is below the 95% target for VLS by 2030 aimed at reducing morbidity, mortality, and the spread of HIV [6]. This study aimed to investigate factors influencing HVL among individuals aged 15 years and older receiving HIV treatment in Tanga City Council, Tanzania. This facility-based cross-sectional study aimed to provide insights into the determinants that impact VLS among PLHIV in the region.

## Materials and methods

### Study setting

This analytical cross-sectional study was conducted at four high-volume care and treatment clinics classified as Tier I in Tanga City Council. The facilities serve about 70% of the PLHIV population in the region (Source: City Medical Officer’s Office, Tanga). Data were collected from the 4^th^ of September to 27^th^ of November 2023, from Tanga Regional Referral Hospital, Ngamiani, Makorora, and Pongwe Health Centers. The total number of PLHIV across all these facilities was 11,374. These facilities serve half of the region’s HIV clients from the 98 of the facilities supported by the U.S. President’s Emergency Plan for AIDS Relief (PEPFAR), therefore offering a representative sample for the study. These facilities provided comprehensive HIV care and treatment services free of charge, including HIV testing, treatment, and VL monitoring, accessible to all individuals.

### Study population

This study involved PLHIV aged 15 years and above, who had been on ART for at least six months, attending the four selected facilities. A systematic random sampling was used to recruit eligible participant who consented to participate in the study through written and verbal consent/assent. Patients with serious health complications or mental challenges were excluded from the study.

### Sample size and sampling

To have a representative sample of PLHIV across the four facilities, the study used a sample size formula for finite populations. The total population of PLHIV in the study area was 11,374, with a reported prevalence of unsuppressed HVL of 6.5% in Tanga [10]. For this study, unsuppressed HVL was defined as a plasma HIV RNA level of ≥1000 copies/mL, in line with WHO guidelines [11]. The sample size was calculated using a 95% confidence level (Z = 1.96), a margin of error of 5%(ε = 0.05), and the known population size [12]. The minimum sample size was 73 for each facility. To account for a 5% non-response rate, the final sample size per facility was adjusted to 77, resulting in a total of 308 PLHIV to be recruited across the four facilities. A systematic sampling approach was utilized to select participants, ensuring representation of PLHIV aged 15 years and older on ART for at least six months. Each clinic day, the CTC appointment register was used to identify eligible clients arriving for services. Based on the expected daily attendance and the required sample size, every second eligible client was approached for participation. When a selected client declined participation, or was ineligible, or was not available after the clinical visit, the next eligible client was invited to participate. A total of 243 out of the estimated sample size of 308 participants were recruited, resulting in a response rate of approximately 78.9%. Data of 233(95.9%) participants with HVL tests conducted were therefore analysed further.

### Data collection procedures

Primary and secondary data sources were used in this study. Primary data were collected using a pretested, interviewer-administered Swahili translated questionnaire, adapted from previous studies  [13,14]. Secondary data were retrieved retrospectively from patients’ records. This included viral load results, history of opportunistic infections, duration on ART, ART regimen, and CTC2 database entries. After being informed about the study objectives, participants who provided written consent were interviewed in Swahili at their Care and Treatment Clinics. Research assistants received training on data collection procedures, participant rights, and rapport building. The questionnaire was pretested at Duga and Tumaini Health Centres to ensure clarity, validity, and reliability. The Content Validity Index (CVI) was 0.86, demonstrating good agreement among experts on the relevance and comprehensiveness of the items. Internal consistency of the multi-item scales was also confirmed, with Cronbach’s alpha values ranging from 0.78 to 0.85, indicating acceptable reliability.

### Study variables

The dependent variable was HVL, defined as a viral load ≥1000 copies/mL based on routine viral load monitoring records documented in the CTC2 database and facility laboratory reports. HVL was categorized as “yes” (coded as 1) for participants with viral load ≥1000 copies/mL and “no” (coded as 0) for those with viral load <1000 copies/mL, independent variables included socio-economic and demographic factors such as age, gender, education, occupation, marital status, smoking status, alcohol consumption [14], stigma (measured through the Abridge Scale) [15], household food insecurity, socio-economic factors (wealth index generated through principle component analysis – PCA)^14^; and clinical data (WHO HIV clinical stage, c-ART type, duration, and presence of opportunistic infections such as Tuberculosis, Cryptococcal infection, and toxoplasmosis. Stigma was measured using the Abridged HIV Stigma Scale, a validated multi-item instrument assessing perceived HIV-related stigma. Responses were scored and summed to generate a total stigma score, which was then categorized into low and high stigma based on predefined thresholds. Food insecurity was assessed using the Household Food Insecurity Access Scale (HFIAS).

### Data analysis

This study employed both descriptive and inferential analyses. Descriptive statistics summarized data using frequencies/counts and mean ± standard deviation (SD) for continuous variables, while percentages were used for categorical variables. The proportion of HVL (>1000 copies/mL) was assessed at a 95% confidence interval and tabulated against participants’ sociodemographic and clinical characteristics using a Pearson Chi-square test. For inferential analyses, multivariate logistic regression estimated the association between independent variables and HVL. Initially, univariate analysis explored unadjusted associations. In the adjusted logistic regression model, a forward stepwise method was used to fit the predictors of HVL where one independent variable was added into a model at a time. The variables which showed multicollinearity (Variance Inflation Factor (VIF)>4) and those with p > 0.2 in the unadjusted model were excluded. Probability > 0.2 were excluded to avoid overfitting the model with variables which might not be significant [16]. However, important sociodemographic and clinical characteristics variables of interest were retained regardless of statistically insignificant in the unadjusted model except those with multicollinearity. Statistical significance was set at p < 0.05. Data analysis was conducted using Stata/MP 14.2 (StataCorp LLC).

### Ethical considerations

Ethical clearance was granted by Muhimbili University of Health and Allied Sciences (DA.282/298/01.C/1878). Permission was obtained from the City Medical Officer and healthcare officials. Written and verbal consent was obtained from participants with an assent for those below 18 years of age. Participants were informed of their rights, including withdrawal at any time without affecting care they receive. Unique identity numbers were used and no personal identifiers were collected. Throughout data collection and analysis we maintained confidentiality. Data was stored on a password-protected computer.

## Results

### Sociodemographic and clinical characteristics of study participants

Majority of participants were aged above 24 years 172(73.8%), the mean age was 32.4 years (±11.4 SD), with females making up 144(61.8%). 134(68.4%) had pre and primary education. Majority of participant were on Combined ART (c-ART) of Tenofovir + Lamivudine + Dolutegravir (TLD) regimen 220 (94.4%) with no participant on HIV clinical stage 4. High HVL was noted among 82(35.2%) [95% CI; 29.3%−41.6%] of the participants with HVL tests results available (Table 1).

**Table 1:** Sociodemographic and clinical characteristics of study participants.

Characteristics		Frequency	Percentage
**Age groups in years**	Young adults 15–24	61	26.2
	Adults >24	172	73.8
**Mean age in years**	32.4 (SD: 11.4)		
**Gender**	Male	89	38.2
	Female	144	61.8
**Education level**	Pre & Primary	134	68.4
	Secondary O-Level	48	24.5
	Secondary A-level and above	14	7.1
**Occupation**	Professional/technical/managerial	11	4.7
	Clerical	8	3.4
	Sales and services	22	9.4
	Skilled manual	27	11.6
	Unskilled manual	47	20.2
	Domestic service	46	19.7
	Agriculture	72	30.9
**Marital status**	Never married	32	13.7
	Married	112	48.1
	Widowed	21	9.0
	Divorced	11	4.7
	Separated	32	13.7
	Cohabiting	25	10.7
**Smoking status**	Never smoked	195	83.7
	Currently smoking	38	16.3
**Alcohol consumption**	Yes	113	48.5
	No	120	51.5
**Stigma status**	Low	56	24.0
	High	177	76.0
F**ood insecurity**	Yes	77	33.0
	No	156	67.0
**Food insecurity severity**	Mild to moderate	113	73.9
	Severe	40	26.1
**Household wealth**	Low	131	56.2
	Medium	67	28.8
	High	35	15.0
**Is distance a big problem**	Yes	19	8.2
	No	214	91.8
**Clinical characteristics**			
**c-ART regimen**	TLD	220	94.4
	Others	13	5.6
**Duration on c-ART**	≤ 12 months	13	5.6
	13-24 months	39	16.7
	≥ 25 months	181	77.7
**c-ART stored in a container**	Yes	207	88.8
	No	26	11.2
**WHO HIV Clinical Stage**	Stage 1	149	63.9
	Stage 2	71	30.5
	Stage 3	13	5.6
	Stage 4	0	0.0
**Opportunistic infection**	Yes	48	20.6
	No	185	79.4
**Viral load**	Low	151	64.8
	High	82	35.2

### Viral load levels by sociodemographic characteristics of the study participants

High HVL prevalence was noted among 32(52.5%) young adults, 14(63.6%) participants from sales and services industry, 6(54.5%) professionals, and 25(53.2%) unskilled labor categories. Additionally, high HVL was prevalent among 59(52.2%) of participants who consume alcohol and 70(39.5%) participants with high level of stigma. Moreover, high HVL was prevalent among 33(82.5%) participants with severe household food insecurity (**Table 2).**

**Table 2:** Viral load by sociodemographic among PLHIV (n = 233).

Variable		Viral load			P-value
		N (%)	N (%)		
		Low 151 (64.8)	High 82 (35.2)		
**Mean age in years**	32.4 (SD: 11.4)				
**Age groups in years**	Young adults 15-24	29 (47.5)		32 (52.5)	<0.001*
	Adults >24	122 (70.9)		50 (29.1)	
**Gender**	Male	55 (62.1)		34 (37.9)	0.478
	Female	96 (66.7)		48 (33.3)	
**Education level(n=196)**	Pre & Primary	82 (61.2)		52 (38.8)	0.335
	Secondary O-Level	31 (64.6)		17 (35.4)	
	Secondary A-level and above	6 (42.8)		8 (57.2)	
**Occupation**	Professional/technical/managerial	5 (45.5)		6 (54.5)	<0.001*
	Clerical	7 (87.5)		1 (12.5)	
	Sales and services	8 (36.4)		14 (63.6)	
	Skilled manual	17 (63.0)		10 (37.0)	
	Unskilled manual	22 (46.8)		25 (53.2)	
	Domestic service	32 (69.6)		14 (30.4)	
	Agriculture	60 (83.3)		12 (16.7)	
**Marital status**	Never married	21 (65.6)		11 (34.4)	0.318
	Married	77 (68.8)		35 (31.2)	
	Widowed	12 (57.1)		9 (42.9)	
	Divorced	9 (81.8)		2 (18.2)	
	Separated	20 (62.5)		12 (37.5)	
	Cohabiting	12 (48.0)		13 (52.0)	
**Smoking status**	Never smoked	130 (66.7)		65 (33.3)	0.178
	Currently smoking	21 (55.3)		17 (44.7)	
**Alcohol consumption**	Yes	54 (47.8)		59 (52.2)	<0.001*
	No	97 (80.8)		23 (19.2)	
**Stigma status**	Low	44 (78.6)		12 (21.4)	0.013*
	High	107 (60.5)		70 (39.5)	
**Food insecurity**	Yes	32 (41.6)		45 (58.4)	<0.001*
	No	119 (76.3)		37 (23.7)	
**Food insecurity severity(n=153)**	Mild to moderate	71 (62.8)		42 (37.2)	<0.001*
	Severe	7 (17.5)		33 (82.5)	
**Household wealth**	Low	75 (57.2)		56 (42.8)	<0.001*
	Medium	42 (62.7)		25 (37.3)	
	High	34 (97.1)		1 (2.9)	
**Distance is a big problem**	Yes	3 (15.8)		16 (84.2)	<0.001*
	No	148 (69.2)		30.8)	

**Note:** Asterisks (*) indicate statistically significant values (P < 0.05).

### Viral load by clinical characteristics of the study participants

Participants on the TLD c-ART regimen had a lower proportion of high HVL 73(33.2%) compared to those on other regimens 9(69.2%, p = 0.008). Those on c-ART for 13–24 months had a higher proportion of high HVL rate of 29(74.4%) than those on c-ART for over 25 months (26.5%, p = 0.001). Proper storage of c-ART significantly reduced high HVL 60(29.0%, p = 0.001). Additionally, individuals in WHO HIV clinical stage three had a higher HVL proportion of 10(76.9%) compared to those in stage one 50(33.6%) and two 22(31.0%, p = 0.005). **Table 3**

**Table 3:** Viral load by clinical factors among PLHIV (n = 233).

Variables		HIV Viral load		P-Value
		Low-N (%)	High-N (%)	
**c-ART regimen**	TLD	147 (66.8)	73 (33.2)	0.008*
	Others	4 (30.8)	9 (69.2)	
**Duration on c-ART**	≤ 12 months	8 (61.5)	5 (38.5)	<0.001*
	13-24 months	10 (25.6)	29 (74.4)	
	≥ 25 months	133 (73.5)	48 (26.5)	
**c-ART stored in a container**	Yes	147 (71.0)	60 (29.0)	<0.001*
	No	4 (15.4)	22 (84.6)	
**WHO HIV Clinical Stage**	Stage 1	99 (66.4)	50 (33.6)	0.005*
	Stage 2	49 (69.0)	22 (31.0)	
	Stage 3	3 (23.1)	10 (76.9)	
**Opportunistic infection**	Yes	31 (64.6)	17 (35.4)	0.971
	No	120 (64.9)	65 (35.1)	

**Note:** Asterisks (*) indicate statistically significant values (P < 0.05).

### Predictors of high viral load among study participants

Several variables met the inclusion criteria for the regression model, having p-values less than 0.2, indicating their potential association with high viral load. These variables included age groups (p = 0.001), alcohol consumption (p = 0.001), stigma status (p = 0.015), food insecurity severity (p = 0.001) and c-ART storage in the container (p = 0.001).

Young adults aged 20–24 were 5.4 times more likely to have a higher HVL than those aged 25 and older (AOR = 5.4, 95% CI: 1.8–15.7, p = 0.002). PLHIV working in sales and services were 6.1 times more likely to have a higher HVL compared to those in agriculture, (AOR = 6.1, 95% CI: 1.1–35.4, p = 0.044). Alcohol consumption increased the likelihood of a higher HVL by 4.2 times compared to non-drinkers (AOR = 4.2, 95% CI: 1.4–12.5, p = 0.011), while those facing high stigma were 4.3 times more likely to have a higher HVL than individuals with good social support and low stigma (AOR = 4.3, 95% CI: 1.0–18.7, p = 0.049). PLHIV experiencing severe food insecurity were 9.6 times more likely to have a higher HVL than those with mild to moderate food insecurity (AOR = 9.6, 95% CI: 2.6–35.2, p = 0.001). Furthermore, improper storage of c-ART outside its original container increased the risk of a higher HVL by 6.7 times compared to those who properly stored their medication (AOR = 6.7, 95% CI: 1.2–38.1, p = 0.033). (**Table 4**)

**Table 4:** Factors Associated with Higher viral load among PLHIV aged ≥15 years.

Characteristics	Viral load	High n %	Univariate		Multivariate	
	Low n%		COR(95% CI)	P value	AOR (95% CI)	P value
**Age groups in years**						
Young adults 15–24	29 (47.5)	32 (52.5)	Reference			
Adults >24	122 (70.9)	50 (29.1)	2.8 [1.6-5.1]	0.001*	5.4 [1.8-15.7]	0.002*
**Sex**						
Female	96 (66.7)	48 (33.3)	Reference			
Male	55 (62.1)	34 (37.9)	1.2 [0.7-2.1]	0.478	1.2 [0.4-3.4]	0.77
**Occupation**						
Agriculture	60 (83.3)	12 (16.7)	Reference			
Professional/technical/managerial	5 (45.5)	6 (54.5)	5.5 [1.5-20.9]	0.012*	7.5 [0.9-60.7]	0.059
Clerical	7 (87.5)	1 (12.5)	0.7 [0.1-5.8]	0.708	1.3 [0.05-33.3]	0.888
Sales and services	8 (36.4)	14 (63.6)	8.7 [3.0-24.6]	0.001*	6.1 [1.1-35.4]	0.044*
Skilled manual	17 (63.0)	10 (37.0)	3.5 [1.4-9.0]	0.008*	8.6 [1.7-43.8]	0.010*
Unskilled manual	22 (46.8)	25 (53.2)	5.7 [2.5-12.9]	0.001*	8.1 [2.0-32.7]	0.003*
Domestic service	32 (69.6)	14 (30.4)	2.6 [1.1-5.7]	0.036*	3.3 [0.7-16.3]	0.138
**Smoking**						
Never smoked	130 (66.7)	65 (33.3)	Reference			
Currently smoking	21 (55.3)	17 (44.7)	1.6 [0.8-3.3]	0.181	0.5 [0.1-2.3]	0.378
**Alcohol consumption**						
No	97 (80.8)	23 (19.2)	Reference			
Yes	54 (47.8)	59 (52.2)	4.6 [2.6-8.3]	0.001*	4.2 [1.4-12.5]	0.011*
**Stigma**						
Low	44 (78.6)	12 (21.4)	Reference			
High	107 (60.5)	70 (39.5)	4.6 [2.6-8.3]	0.015*	4.3 [1.0-18.7]	0.049*
**Food insecurity severity**						
Mild to moderate	71 (62.8)	42 (37.2)	Reference			
Severe	7 (17.5)	33 (82.5)	8.0 [3.2-19.6]	0.001*	9.6 [2.6-35.2]	0.001*
**Clinical factors**						
**c-ART stored in the container**						
Yes	147 (71.0)	60 (29.0)	Reference			
No	4 (15.4)	22 (84.6)	11.6[3.8-34.8]	0.001*	6.7 [1.2-38.1]	0.033*

Note: COR – crudes odds ratio, AOR – Adjusted odds ratio

Asterisks (*) indicate statistically significant values (P < 0.05, OR>1)

## Discussion

This study found a high HVL prevalence of 35.2% [95% CI; 29.3%−41.6%] among PLHIV aged 15 and older on c-ART for at least 6 months. Higher proportions were found in young adults, varied with occupations, individuals experiencing stigma, those from severe food insecurity households, from households with low wealth index, traveling long distances to health facilities, those on non-TLD regimens, those on c-ART for 12–24 months, and with advanced WHO HIV stages. Similar studies in Rwanda and Cameroon reported a prevalence of 29% and 20.6% [13,17], particularly among young adults and those on c-ART for 12–24 months.

This study found that severe food insecurity is associated with elevated VL among PLHIV. Alongside c-ART, a balanced diet is essential for improving health and nutritional status. These findings align with previous research linking food insecurity to higher VL, highlighting the importance of ensuring food security and access to essential nutrients for vulnerable populations [18]. Hunger can lead to medication discontinuation and reduced care attendance, negatively affecting adherence and contributing to psychological disorders.

Participants with high stigma levels in this study were more likely to have high HVL, likely due to poor communication with healthcare providers and reduced treatment adherence. Stigma may also worsen mental health issues like depression [19], contributing to treatment fatigue. A study in Dar es Salaam and other contexts found similar effects of stigma on VLS [20,21],. suggesting that addressing stigma and psychological issues could reduce HVL and prevent treatment failure.

Alcohol consumption, especially binge drinking, can impair judgment regarding medication adherence and increase engagement in high-risk sexual behaviours, leading to poor adherence and new HIV infections [22], which may result in persistently high HVL. This study’s findings align with research from Rwanda, showing significantly higher odds of high HVL among alcohol consumers [13]. Similarly, previous studies have found a strong association between alcohol use and increased odds of high HVL among PLHIV on c-ART [23]. Providing intensive counselling among PLHIV on alcohol cessation or moderation can help reduce HVL and the risk of treatment failure.

This study found that young adults had significantly higher odds of high viral loads compared to older participants, likely due to their relatively lower capacity to mobilize resources for ongoing care. Factors such as cognitive and physical development, mental health, substance use, and family support can impact treatment adherence among adolescents and young adults [13]. The Tanzania HIV Impact Survey (2022/23) indicated that younger adults were less likely to achieve VLS than their adult counterparts. These findings align with studies in Botswana [24] and the U.S. [25], which also showed that younger adults struggle more with treatment adherence and achieving viral load suppression compared to older adults.

Studies shows occupation significantly impacts medication adherence and VLS among PLHIV [7,26]. The study found that participants working in sales, skilled manual, and unskilled manual occupations were more likely to have elevated HVL. This may be due to tight work schedules and concerns about job security, which can limit their ability to attend clinic appointments and adhere consistently to ART.

Studies have reported conflicting results whereby a study in Ghana did not found a link between employment and HVL [27], while a study in Nigeria suggested unemployment correlates with better VLS [28]. Furthermore, a meta-analysis study indicated that employed individuals generally adhere better to treatment [29]. These conflicting results highlight the complexity of the relationship between employment status and VLS among PLHIV. Nonetheless, it is essential to consider occupation as a potential influencing factor when initiating c-ART for people living with HIV.

### Strength and limitation

This study has several strengths, including its focus on PLHIV in Tanga, Tanzania, the use of probability sampling, and standardized data collection tools, which enhance the relevance and reliability of the findings. The study also provides important insights into the influence of stigma, food insecurity, and substance use on viral load suppression among PLHIV. However, several limitations should be acknowledged. The cross-sectional design limits the ability to establish causal relationships between variables. The study relied on self-reported information, which may be subject to recall and social desirability bias. Viral load measurements were based on a single time point, which may not fully capture longitudinal treatment outcomes. The response rate was 78.9%, with non-participation due to absent clients, ineligibility, or refusal, which may introduce selection bias. Some adjusted odds ratios had wide confidence intervals, likely due to small cell counts or limited sample sizes, reducing estimate precision. Finally, the measurement of economic status may not have fully captured participants’ socioeconomic conditions.

### Conclusion and recommendation

The high prevalence of elevated viral load among PLHIV on c-ART for six months is a concern, with 1 in 3 at risk of treatment failure. Key predictors include sociodemographic factors, food insecurity, stigma, alcohol use, and employment. Targeted interventions like food availability support and stigma reduction are vital.

## Abbreviation:

AIDS: Acquired immune deficiency syndrome
AOR: Adjusted odds ration
ART: Antiretroviral therapy
c-ART: Combined ART
CI: Confidence interval
CTCs: Care and Treatment Centre
HVL: HIV Viral Load
PCA: Principle Component Analysis
PEPFAR: U.S. President’s Emergency Plan for AIDS Relief
PLHIV: People living with HIV
SD: Standard deviation
TLD: Tenofovir + Lamivudine + Dolutegravir
UNAIDS: United Nations Programme on HIV/AIDS
VIF: Variance Inflation Factor
VLS: Viral Load Suppression.
