# Supplementary material for: Factors associated with high viral load among HIV clients aged 15 years and older receiving treatment in Tanga city council, Tanzania: A facility-based cross-sectional study
Source: PLoS One. 2026 May 7;21(5):e0348862. doi: 10.1371/journal.pone.0348862 (PMC13152164; doi:10.1371/journal.pone.0348862)
Supplement: Attachment — Submitted filename: Reviewer comments_HIGH VIRAL LOAD FACTORS_PLOS ONE.pdf [file pone.0348862.s002.pdf]

Factors associated with high viral load among HIV clients aged 15 years and older receiving treatment in Tanga city council, Tanzania: A facility-based cross-sectional study

Thank you for the opportunity to review the manuscript titled: **Factors associated with high viral load among HIV clients aged 15 years and older receiving treatment in Tanga city council, Tanzania: A facility-based cross-sectional study**

Some minor comments;

Abstract

This part is well written and contains all the elements of a good abstract, the following are minor correction to make:

1. In the background part of the abstract: Line 4 add the word "years" after "15"
2. Method part of abstract: Recheck and clarify the correct long form of CTC as authors have written it as care and testing center but from what I know is care and treatment centre (or clinic). Also the author need to add a statement specifying that the clinical data were collected from patient's record to be clear to the reader.
3. Conclusion part of abstract: The author recommended about stigma but this was not reported anywhere in the result section.

Background:

This is also well written, but minor corrections to make in the following parts

4. Paragraph 2 Line 2: Add the word "years" after "15"
5. Paragraph 2 Line 3: What is the long form of VL? (Recommended all abbreviation to be written in full in their first mention with abbreviation in brackets, then continue with abbreviation in the subsequent use of the term)
6. Paragraph 2 Line 4: Which practice is being referred here? Please specify.
7. Paragraph 3 Line 2: Add the word "year" before "2019", and the authors are recommended to use updated reference for example there are data for year 2022, check and correct word spacing throughout the document.
8. Paragraph 3 Line 2: What is the long form of HVL? (Recommended all abbreviation to be written in full in their first mention with abbreviation in brackets, then continue with abbreviation in the subsequent use of the term).

Methods:

By and large this section is okay, except for minor comments under the following parts:

Study setting

9. Line 3: The author need to correct the word "of" which is written in superscript back to normal

10. Line 7: The sentence "all facilities provided comprehensive HIV…………………………….." Is not clear to the reader, please rephrase.

Study population

11. Line 1: suggested the first sentence to begin as "This study involved PLHIV aged 15 years and above, who had……………………..".

Data sources, collection and Tools

12. Suggested the subheading (**Data sources, collection and Tools)** to be changed and written as **"Data collection procedure"**
13. Line 1: Change the words "in the study" to be "in this study"
14. Line 2: Add words "Swahili translated" before the word "questionnaire"
15. Line 3: Replace the word "time" with the word "duration"
16. Line 5: Suggested, the sentence "After identifying participants……" to be rephrased and written as "identified participants were interviewed using Swahili translated questionnaire………"
17. Line 8: The sentence "The English questionnaire was translated……………." is not needed here as the information has already been said above, so suggested to delete it.

Study variables

18. Recommended all abbreviation to be written in full in their first mention with abbreviation in brackets, then continue with abbreviation in the subsequent use of the term
19. The word "HAART" sounds ancient terminology nowadays the word combined antiretroviral therapy (c-ART) is used, so suggested to replace the word "HAART" with "c-ART" throughout the document

Data analysis

20. The author need to describe the test used to compare proportions (low versus high viral load) for the results presented in table 1 and 2
21. Check and correct word spacing

Ethical considerations

22. The subheading to be re-written as "Ethical considerations" (with an "s" added on word "consideration")
23. Lin 3: For the participants under 18 years normally seek for assent and not consent. Please re-check and correct accordingly.
24. Line 4: Did withdrawal from the study affected their cate at the CTC? if not suggested to add a statement "without affecting care they receive" after the word "time"

Results

This is well written, but the following are minor comments for improvement:

25. The author is advised to add another table as table 1 with the table title "Sociodemographic and clinical characteristics of study participants" which will have 3 column as exemplified below:

Characteristics	Frequency (n)	Percentage (%)

This will give a general picture of characteristics of participants involved in this study.

Viral load by sociodemographic characteristics of the respondents

26. Suggested the subheading to be re-written as "**Viral load levels by sociodemographic characteristics of the study participants"**

27. I don't think logistic regression analysis was used to determine the significance of difference in proportion of viral load level between variables. The author need to re-check and clarify this.

Viral load by clinical characteristics of the study participants

28. Check word spacing and correct
29. Indicate the p-value in brackets for all variables that showed significant difference

Predictors of high viral load among study participants

30. In statement form, first write the independent variables related to your outcome variables on Univariate. Then, on Multivariate, write the factors related to your outcome variable (higher viral load) by interpreting the association using AOR and CI (as described currently)

31. Table 3: Amend the table column heading as suggested below:

Characteristics	Viral load		Univariate		Multivariate	
	Low n (%)	High n (%)	COR (95% CI)	P value	AOR (95% CI)	P value

This will make the reader to clearly follow and understand

32. Make OR under the univariate "COR" and multivariate "AOR" in table 3, and write the whole form as footnotes beneath the tables

33. There is no p value with asterisk (*) in the table 3 rather bolded p value. If you use asterisk (*) , this will better be presented as footnote beneath the table in the following format: "

    *Statistically significant

Discussion

34. Paragraph 1 line 1: Present prevalence with CI in brackets
35. Paragraph 1 line 4: repace “late WHO HIV stage” with “advanced HIV stages”
36. Paragraph 6 line 1: suggested to rephrase the sentence “Literature shows mixed findings……………………………..” re-writing as “Studies have reported conflicting results whereby a study in Ghana did not found a link between employment and HVL while a study in Nigeria suggested unemployment correlates with better VLS. Furthermore, a meta-analysis study indicated that employed individuals generally adhere better to treatment.”

Conclusion and recommendation

37. Line 1: Change the words “is concerning” to be “is a concern”

38. Recommendations and conclusions should be grounded in the findings of your study, particularly when recommending any accountable body, which should be based on factors related to your outcome variable (high viral load).

Grammar and missing/repeated words

39. Please check over grammar, spelling, word spacing and missing/duplicated words throughout the document and correct.
40. The manuscript is not numbered line-by-line in order to provide simple peer-review, please add this for re-submission
