# Supplementary material for: Factors associated with high viral load among HIV clients aged 15 years and older receiving treatment in Tanga city council, Tanzania: A facility-based cross-sectional study
Source: PLoS One. 2026 May 7;21(5):e0348862. doi: 10.1371/journal.pone.0348862 (PMC13152164; doi:10.1371/journal.pone.0348862)
Supplement: Attachment — Submitted filename: 13NOV 25-PONE-D-25-20662 HIV in Tanzania.docx [file pone.0348862.s001.docx]

**The Editor**
**PLOS ONE Journal**

**13 November 2025**

**RE: Reviewer Comments to the Author on**
*Factors associated with high viral load among HIV clients aged 15 years and older receiving treatment in Tanga city council, Tanzania: A facility-based cross-sectional*
**(Manuscript ID: PONE-D-25-20662)**

**Dear PLOS ONE Editor,**

I would like to thank the authors for their contribution to this important topic. The manuscript addresses factors associated with high viral load among HIV clients aged 15 years and older receiving treatment in Tanga City Council, Tanzania, using a facility-based cross-sectional design. The study is relevant and provides valuable insights for HIV management and public health strategies in the region.

Below, I provide detailed reviewer comments to improve the clarity, rigor, and overall quality of the manuscript.

**Reviewer Comments**

**1. Abstract**
Please add a heading titled **“Aim”** in the manuscript to clearly state the main objective of the study.

**2. Materials and Methods: Study Setting**
The manuscript states that the facilities serve about 70% of the PLHIV population in the region. Please provide a reference to support this statement.

**3. Study Population / Inclusion Criteria**
Please provide more details on how study participants were recruited. If convenient, clarify the informed consent process, specifically, whether consent was obtained verbally or in writing.

**4. Sample Size and Sampling
4.1** The manuscript mentions a total population of 11,374 PLHIV and a 6.5% prevalence of unsuppressed high viral load (HVL) in Tanga. Please kindly define the threshold or level of viral load used to categorize it as “unsuppressed.

**4.2** **Sample Size Inconsistency**
The manuscript states that a total of 308 PLHIV were to be recruited across the four facilities, but the Methods section and the Abstract subheading mention 243 participants. Please clarify this discrepancy to ensure consistency.

**5. Data Sources, Collection, and Tools**
The manuscript states that the English questionnaire was translated into Swahili for clarity and pretested at non-research sites (Duga and Tumaini Health Centres) to ensure validity and reliability. Could the authors clarify whether any multi-item scales were included in the questionnaire? If so, please provide the Cronbach’s alpha values or other reliability statistics to demonstrate internal consistency**.**

**6. Data Analysis**
**6.1** The manuscript defines high viral load (HVL) as a viral load greater than 1000 copies/dL. Please clarify the units used, as the standard reporting for HIV viral load is in copies/mL, not copies/dL

**6.2 .** The manuscript states that a stepwise method was used in the multivariable logistic regression to identify predictors of high viral load. Please clarify which stepwise approach was applied (forward, backward, or both), and specify the criteria for variable entry and removal (e.g., p-value thresholds or information criteria) to enhance transparency and reproducibility of the analysis.

**6.3** Please mention the full term *Variance Inflation Factor (VIF)* before using the abbreviation. Also, clarify the rationale for excluding variables with p > 0.2 in the unadjusted model.

**7. Tables 1, 2, and 3**
It is recommended to add a footnote indicating that *p* ≤0.05 was considered statistically significant for the variables in the tables. This will help clarify the interpretation of significant results for readers.

**7.1 Table 2**

The results describe high viral load proportions for WHO clinical stages two and three, but stage four is not mentioned. Please clarify whether data for stage four were unavailable, excluded, or not applicable, and if available.

**7.2 Table 2**

Please define the abbreviations ‘HAART’ and ‘TLD’ at their first occurrence in a footnote, to ensure clarity and consistency for readers unfamiliar with these terms.

**7.3 Table 3 (Regression Model)**
Please clarify the criteria used to select variables included in the regression model for Table 3, titled “Factors Associated with Higher Viral Load among PLHIV aged ≥15 years.” Specify whether inclusion was based on statistical significance in univariate analysis, theoretical relevance, or other selection criteria.

**7.4 Table 3**

Please remove the repeated word “Note” in the footnote under Table 3.

**8. Headings** Please add the following headings to the manuscript to meet journal requirements and enhance clarity and completeness:

- **Authors’ Contributions**
- **List of Abbreviations** (in alphabetical order)

**Summary and Recommendation**
Thank you for considering my revisions.

This manuscript, “Factors Associated with High Viral Load among HIV Clients Aged 15 Years and Older Receiving Treatment in Tanga City Council, Tanzania: A Facility-Based Cross-Sectional Study,” addresses an important and timely topic with clear relevance to HIV program improvement and public health practice. However, minor revisions are required to meet academic and journal standards fully.

The suggested improvements will enhance clarity, methodological transparency, and overall presentation.

**Recommendation:** Minor Revision

Best regards,
**Zainab Mahmood Al-Zadjali**

Reviewer, Department of Family Medicine and Public Health, College of Medicine and Health Sciences, Sultan Qaboos University, Oman
