# Supplementary material for: Factors associated with high viral load among HIV clients aged 15 years and older receiving treatment in Tanga city council, Tanzania: A facility-based cross-sectional study
Source: PLoS One. 2026 May 7;21(5):e0348862. doi: 10.1371/journal.pone.0348862 (PMC13152164; doi:10.1371/journal.pone.0348862)
Supplement: Attachment — Submitted filename: Responses to reviewers comments AKKIE-BS 20.03.206.docx [file pone.0348862.s003.docx]

**Response to Reviewers: A Matrix**

We are very thankful for reviewers for reviewing our work. We agree with all reviewers comments and have worked on them which has improved greatly our manuscript. The point by point response to all reviewers comments is listed in the matrix below.

**Reviewer 1**

| **S.N** | **Reviewers Comments** | **Response** | **Page** |
| --- | --- | --- | --- |
| **Abstract** | | | |
| 1 | In the background part of the abstract: Line 4 add the word “years” after “15” | Thank you, the word year has been added. | Line 15 |
| 2 | Method part of abstract: Recheck and clarify the correct long form of CTC as authors have written it as care and testing center but from what I know is care and treatment centre (or clinic). Also the author need to add a statement specifying that the clinical data were collected from patient’s record to be clear to the reader. | Thank you for the comment. We have reviewed the manuscript throughout and change into “Care and Treatment Centre (CTC)” | Line 23 and throughout |
| 3 | Conclusion part of abstract: The author recommended about stigma but this was not reported anywhere in the result section. | Thank you, However, stigma is reported in Table 1 and table 4. In table 4 it is shown to be associated with HVL and in the abstract result section. | Table 1 and 4 |
| **Background** | | | |
| 4 | Paragraph 2 Line 2: Add the word “years” after “15” | Thank you, have added “years” on this line | Page 3 – Line 64 |
| 5 | Paragraph 2 Line 3: What is the long form of VL? (Recommended all abbreviation to be written in full in their first mention with abbreviation in brackets, then continue with abbreviation in the subsequent use of the term) | Thank you, a definition added in the commented section as “ HIV Viral Load (HVL)”. All other abbreviations have been defined when used for the first time. | Page 3 (Line 65) and throughout |
| 6 | Paragraph 2 Line 4: Which practice is being referred here? Please specify. | Thank you, we have specified the document that recommends this in Tanzania settings which is Tanzanian HVL testing guideline.. | Page 3 (Line 66 – 67) |
| 7. | Paragraph 3 Line 2: Add the word “year” before “2019”, and the authors are recommended to use updated reference for example there are data for year 2022, check and correct word spacing throughout the document. | Thank you, the word “year” has been added | Page 3 (Line 71) |
| 8. | Paragraph 3 Line 2: What is the long form of HVL? (Recommended all abbreviation to be written in full in their first mention with abbreviation in brackets, then continue with abbreviation in the subsequent use of the term). | Thank you, the HVL has been defined as HIV Viral Load in paragraph 4 | Page 3 (Line 81) |
|  | **Methods** |  |  |
|  | **Study setting** |  |  |
| 9 | Line 3: The author needs to correct the word “of” which is written in superscript back to normal | Thank you, the of in the superscript has be removed and written normally | Page 4 (Line 88) |
| 10 | Line 7: The sentence “all facilities provided comprehensive HIV…………………………….” Is not clear to the reader, please rephrase. | Thank you, we have edited to “These facilities provided comprehensive HIV care and treatment services free of charge, including HIV testing, treatment, and VL monitoring, accessible to all individuals”. | Page 4 (Line 93) |
| 11 | Study population  Line 1: suggested the first sentence to begin as “This study involved PLHIV aged 15 years and above, who had……………………..”. | Thank you, we have edited to “This study involved PLHIV aged 15 years and above, who had been on ART for at least six months, attending the four selected facilities” | Page 4 (Line 97 – 98) |
|  | **Data sources, collection and Tools** |  |  |
| 12 | Suggested the subheading (**Data sources, collection and Tools)** to be changed and written as **“Data collection procedure”** | Thank you, we agree with the reviewer and have edited the sub-headings into **“*Data collection procedures”*** | Page 5 (Line 118) |
| 13 | Line 1: Change the words “in the study” to be “in this study” | Thank you for the comment. We have edited the sentence into *“in this study”* | Page 5 (Line 119) |
| 14 | Line 2: Add words “Swahili translated” before the word “questionnaire” | Thank you, we have re-written the text as *“the Swahili translated questionnaire”* as per the comment | Page 5 (Line 120) |
| 15 | Line 3: Replace the word “time” with the word “duration” | Thank you, we have replaced time with duration as suggested | Page 5 (Line 122) |
| 16 | Line 5: Suggested, the sentence “After identifying participants……” to be rephrased and written as “identified participants were interviewed using Swahili translated questionnaire………” | Thank you, based on the reviewers comments, the paragraph has been rewritten. | Page 5(Line 119 – 130) |
| 17 | Line 8: The sentence “The English questionnaire was translated………………” is not needed here as the information has already been said above, so suggested to delete it. | Thank you, based on the reviewers comments, the paragraph has been rewritten. | Page 5 (Line 119 – 130) |
|  | **Study variables** |  |  |
| 18 | Recommended all abbreviation to be written in full in their first mention with abbreviation in brackets, then continue with abbreviation in the subsequent use of the term | Thank you for the comment. We have defined the abbreviations when first in use throughout the manuscript | Througout the manuscript |
| 19 | The word “HAART” sounds ancient terminology nowadays the word combined antiretroviral therapy (c-ART) is used, so suggested to replace the word “HAART” with “c-ART” throughout the document | Thank you, we agree with the reviewer and have changed the outdated HAART to c-ART throughout the manuscript | Throughout the manuscript. |
|  |  |  |  |
|  | **Data analysis** |  |  |
| 20 | The author needs to describe the test used to compare proportions (low versus high viral load) for the results presented in table 1 and 2 | Thank you, have added a Chi – Square test which was used. | Page 6 ( Line 150) |
| 21 | Check and correct word spacing | Thank you for this observation. We have checked throughout the manuscript for spaces and other typos. | Throughout |
|  | **Ethical considerations** |  |  |
| 22 | The subheading to be re-written as “Ethical considerations” (with an “s” added on word “consideration”) | Thank you, we have edited accordingly and added “s” at the end of consideration. | Page 6 ( Line 161) |
| 23 | Line 3: For the participants under 18 years normally seek for assent and not consent. Please re-check and correct accordingly. | Thank you for the comment. For the minors who were below 18 years, an assent was requested. We have added this under the ethical sub section. | Page 6 (Line 164) |
| 24 | Line 4: Did withdrawal from the study affected their cate at the CTC? if not suggested to add a statement “without affecting care they receive” after the word “time” | Thank you, we have added this since and now reads “Participants were informed of their rights, including withdrawal at any time without affecting care they receive”……it did not affect their service if they declined to participate | Page 6 ( Line 165 – 166) |
| 25 | The author is advised to add another table as table 1 with the table title “Sociodemographic and clinical characteristics of study participants” which will have 3 columns as exemplified below: Characteristics \| Frequency (n) \| Percentage (%) | Thank you, we have added this table, and now reads as “Table 1” | Table 1 |
|  | **Viral load by sociodemographic characteristics of the respondents** |  |  |
| 26 | Suggested the subheading to be re-written as “**Viral load levels by sociodemographic characteristics of the study participants”** | Thank you, we have adopted this heading | Page 8 (Line 181) |
| 27 | I don’t think logistic regression analysis was used to determine the significance of difference in proportion of viral load level between variables. The author needs to re-check and clarify this. | Thank you, we have added Chi-square test that was used for in this data analysis section of the methodology | Page 6 ( Line 150) |
| **Viral load by clinical characteristics of the study participants** | | | |
| 27 | Check word spacing and correct | Thank you. We have checked throughout the manuscript and edited along to ensure spaces are correct. | Throughout the manuscript |
| 28 | Indicate the p-value in brackets for all variables that showed significant difference | Thank you, p-values are now added. | Page 10(Line 192 – 197) |
| **Predictors of high viral load among study participants** | | | |
| 29 | In statement form, first write the independent variables related to your outcome variables on Univariate. Then, on Multivariate, write the factors related to your outcome variable (higher viral load) by interpreting the association using AOR and CI (as described currently) | Thank you. We have added the description for the univariate analysis and then multivariate as suggested in the text. | Page 10 – 11( Line 202 – 2016) |
| 30 | Table 3: Amend the table column heading as suggested below: Characteristics Viral load Univariate Low n (%) High n (%) COR (95% CI) P value Multivariate AOR (95% CI) P value This will make the reader to clearly follow and understand | Thank you very much. The table has been amended as suggested. | Table 4 |
|  | Make OR under the univariate "COR" and multivariate "AOR" in table 3, and write the whole form as footnotes beneath the tables | Thank you, we have edited and added the whole form as a footnote | Table 4 |
| 31 | There is no p value with asterisk (*) in the table 3 rather bolded p value. If you use asterisk (*) , this will better be presented as footnote beneath the table in the following format: “ *Statistically significant | Thank you for the comment. We have added the asterisk to all tables with p-values and the footnotes beneath the table. | Table 2- 4 |
|  | Discussion |  |  |
| 32 | Paragraph 1 line 1: Present prevalence with CI in brackets | Thank you, CI added in the brackets | Page 12 ( Line 221) |
| 33 | Paragraph 1 line 4: repace “late WHO HIV stage” with “advanced HIV stages” | Thank you. We have edited to advance ----and with advanced WHO HIV stages | Page 12 (Line 225) |
| 34 | Paragraph 6 line 1: suggested to rephrase the sentence “Literature shows mixed findings……………………………..” re-writing as “Studies have reported conflicting results whereby a study in Ghana did not found a link between employment and HVL while a study in Nigeria suggested unemployment correlates with better VLS. Furthermore, a meta-analysis study indicated that employed individuals generally adhere better to treatment.” | Thank you, edited as suggested –“Studies have reported conflicting results whereby a study in Ghana did not found a link between employment and HVL^27^, while a study in Nigeria suggested unemployment correlates with better VLS^28^. Furthermore, a meta-analysis study indicated that employed individuals generally adhere better to treatment “ | Page 13 ( Line 259 – 262) |
|  | **Conclusion and recommendation** |  |  |
| 35 | Line 1: Change the words “is concerning” to be “is a concern” | Thank you, edited to is a concern …..The high prevalence of elevated viral load among PLHIV on c-ART for six months is a concern, with 1 in 3 at risk of treatment failure. | Page 14 (Line 280 – 281) |
| 36 | Recommendations and conclusions should be grounded in the findings of your study, particularly when recommending any accountable body, which should be based on factors related to your outcome variable | Thank you. We agree with the reviewer’s comment, and have removed variables which were not in the results | Page 14 ( Line 282 – 283) |
|  | **Grammar and missing/repeated words** |  |  |
| 37 | Please check over grammar, spelling, word spacing and missing/duplicated words throughout the document and correct. | Thank you, all have been checked and complied to. | Through out |
| 38 | The manuscript is not numbered line-by-line in order to provide simple peer-review, please add this for re-submission | Thank you, this has been added in this submission |  |

**Reviewer 2**

| **NO.** | **Comment** | **Response** | **Page** |
| --- | --- | --- | --- |
|  | Abstract |  |  |
| 1. | **Abstract** Please add a heading titled **“Aim”** in the manuscript to clearly state the main objective of the study. | Thank you, the aim has been added added | Line 19 |
| 2. | **Materials and Methods: Study Setting** The manuscript states that the facilities serve about 70% of the PLHIV population in the region. Please provide a reference to support this statement | Thank you for the comment. The statement that the selected facilities serve approximately 70% of the PLHIV population in the region is based on administrative reports from the City Medical Officer’s Office (CMO), Tanga, Tanzania, which oversees HIV care and treatment services in the municipality. The manuscript has been revised to clarify this source (Source: City Medical Officer’s Office, Tanga). | Page 4 (Line 88) |
| 3 | **Study Population / Inclusion Criteria** Please provide more details on how study participants were recruited. If convenient, clarify the informed consent process, specifically, whether consent was obtained verbally or in writing | Thank you. We have added the details that participants were recruited through systematic sampling of eligible participants. Also, we have added how consent was obtained through both written and verbal consenting. The details are also provided in the sampling size and ethical clearance sections. | Page 4 and 6 |
| 4.1 | **Sample Size and Sampling 4.1** The manuscript mentions a total population of 11,374 PLHIV and a 6.5% prevalence of unsuppressed high viral load (HVL) in Tanga. Please kindly define the threshold or level of viral load used to categorize it as “unsuppressed. | Thank you for pointing this out. In our study, “unsuppressed high viral load (HVL)” is defined as a plasma HIV RNA level of ≥1000 copies/mL, consistent with the World Health Organization guidelines for virologic suppression. | Page 4 (Line 104 – 105) |
| 4.2 | **Sample Size Inconsistency** The manuscript states that a total of 308 PLHIV were to be recruited across the four facilities, but the Methods section and the Abstract subheading mention 243 participants. Please clarify this discrepancy to ensure consistency. | Thank you very much, the calculated sample size was 308. However, 243 participated in the study of which 233 had the HVL test conducted. Our analysis is based on 233 who had the test done. | Page 4 ( Line 109 – 117) |
| 5 | **Data Sources, Collection, and Tools** The manuscript states that the English questionnaire was translated into Swahili for clarity and pretested at non-research sites (Duga and Tumaini Health Centres) to ensure validity and reliability. Could the authors clarify whether any multi-item scales were included in the questionnaire? If so, please provide the Cronbach’s alpha values or other reliability statistics to demonstrate internal consistency**.** | We thank the reviewer for this important observation. Yes, the questionnaire included several multi-item scales adapted from previous studies to assess participant knowledge, attitudes, and practices. The **Content Validity Index (CVI) was 0.86,** indicating good expert agreement on the relevance and comprehensiveness of the items. Internal consistency of the scales was also assessed, with **Cronbach’s alpha values ranging from 0.78 to 0.85**, demonstrating acceptable reliability. | Page 5 (Line 127 – 130) |
| 6.1 | **Data Analysis**  **6.1** The manuscript defines high viral load (HVL) as a viral load greater than 1000 copies/dL. Please clarify the units used, as the standard reporting for HIV viral load is in copies/mL, not copies/dL | We thank the reviewer for pointing this out. This was a typographical error in the manuscript. The correct unit for defining high viral load (HVL) is copies/mL, consistent with standard HIV reporting guidelines. Accordingly, HVL in our study is defined as a viral load of ≥1000 copies/mL. The manuscript has been corrected to reflect this. | Page 4 (Line 104 -105) |
| 6.2 | The manuscript states that a stepwise method was used in the multivariable logistic regression to identify predictors of high viral load. Please clarify which stepwise approach was applied (forward, backward, or both), and specify the criteria for variable entry and removal (e.g., p-value thresholds or information criteria) to enhance transparency and reproducibility of the analysis. | A forward stepwise method has been added to clarify the way independent variables were added into the model. | Page 6 (Line 151 – 158) |
| 6.3 | Please mention the full term *Variance Inflation Factor (VIF)* before using the abbreviation. Also, clarify the rationale for excluding variables with p > 0.2 in the unadjusted model. | Thank you, we have defined the VIF. We have also added the reason for using the probability > 0.2 to exclude variables in adjusted model which is to avoid overfitting the model with variables which might not be significant | Page 6 ( Line 154 – 156) |
| 7. | **Tables 1, 2, and 3** It is recommended to add a footnote indicating that *p* ≤0.05 was considered statistically significant for the variables in the tables. This will help clarify the interpretation of significant results for readers | Thank you, we have added a note at the end of each table to indicate the significance at 5% with asterisk to all variables values which were statistically significant. | Table 2-4 |
| 7.1 | **Table 2**  The results describe high viral load proportions for WHO clinical stages two and three, but stage four is not mentioned. Please clarify whether data for stage four were unavailable, excluded, or not applicable, and if available. | Thank you, in our sample we did not have any participant with this stage. We have added this in the participant characteristics Table 1. | Table 1 |
| 7.2 | **Table 2**  Please define the abbreviations ‘HAART’ and ‘TLD’ at their first occurrence in a footnote, to ensure clarity and consistency for readers unfamiliar with these terms. | Thank you, the HAART has been replaced with c-ART throughout the document, TLD has be defined in its first mentioning. | Page 7 ( Line 175) |
| 7.3 | **Table 3 (Regression Model)** Please clarify the criteria used to select variables included in the regression model for Table 3, titled “Factors Associated with Higher Viral Load among PLHIV aged ≥15 years.” Specify whether inclusion was based on statistical significance in univariate analysis, theoretical relevance, or other selection criteria. | Thank you, the variable were included based on their having a p value ≤0.2 in univariate analysis and based on their relevance. | Page 6 ( Line 154 – 156) |
| 7.4 | **Table 3**  Please remove the repeated word “Note” in the footnote under Table 3. | Thank you, the word is removed | Table 4 |
| 8 | **Headings** Please add the following headings to the manuscript to meet journal requirements and enhance clarity and completeness:   - **Authors’ Contributions** - **List of Abbreviations** (in alphabetical order) | Thank you, contribution of the authors added and List of abbreviation added. | 18,19 |

**Academic Editors Comments**

| **Study design and analysis inconsistencies** | Response | Page |
| --- | --- | --- |
| The manuscript states inclusion of adults **≥15 years**, yet regression analysis uses a “20–24” category, skipping 15–19 years—unclear if 15–19 were excluded or combined elsewhere | Thank you, the 15 – 24 category was used as a reference category, it’s now included in the regression table (Table 3) | Table 4 |
| Reporting of logistic regression is inconsistent: variables retained “regardless of significance” conflict with the stepwise exclusion logic described | We thank the reviewer for this observation. To clarify, variables with p ≤ 0.20 in the unadjusted analysis were considered for inclusion in the forward stepwise multivariable logistic regression model to reduce the risk of overfitting. However, some key sociodemographic and clinical variables identified a priori based on literature and study objectives were retained in the model regardless of their unadjusted statistical significance, provided they did not exhibit multicollinearity (VIF > 4). The manuscript has been revised to clearly explain this modelling strategy. | Page 6 ( Line 154 – 156) |
| **Sampling and sample sizes issues** |  |  |
| Initial sample size was **308**, but only **243** were enrolled (78.9% response). Reasons for non‑participation and implications for bias are not discussed | We thank the reviewer for this observation. A total of 243 participants were enrolled from the initially estimated sample size of 308, resulting in a response rate of 78.9%. Non-participation was primarily due to absent clients, ineligibility, or refusal to participate at the time of data collection. This may introduce selection bias, as those who did not participate could differ in clinical or sociodemographic characteristics from those who were included. This limitation has been acknowledged in the discussion to highlight its potential impact on the generalizability and representativeness of the study findings. | Page 4 ( Line 109 – 117) |
| Systematic sampling approach is insufficiently described (sampling interval, starting point, handling of absent clients). | We thank the reviewer for this comment. The Methods section has been revised to provide a clearer description of the systematic sampling procedure, including how eligible clients were identified from the CTC attendance register, how the sampling interval was applied during clinic visits, and how cases of absent, ineligible, or declining participants were handled by approaching the next eligible client. These clarifications have been added to improve transparency of the sampling process. | Page 4 ( Line 109 - 115) |
| **Outcome definition inconsistency** |  |  |
| VL >1000 copies is defined as high viral load, but tables sometimes use “greater than 1000 copies/dl”—a nonstandard unit that must be corrected. | We thank the reviewer for noting this inconsistency. The use of “copies/dL” in some tables was a typographical error. The correct unit is copies/mL, and this has been corrected throughout the manuscript and tables. | Throughout the manuscript |
| **Potential misclassification of variables** |  |  |
| Time on ART categories (≤12 months, 13–24, ≥25 months) are used, but it is unclear whether VL tests were first, routine, or repeat measures | We thank the reviewer for this comment. The manuscript has been revised to clarify that viral load measurements were obtained from routine viral load monitoring records in the CTC2 database and from facility laboratory records. These measurements may include routine or repeat viral load tests conducted as part of standard HIV care and treatment services. This clarification has been added to the Methods section. | 6 |
| Food insecurity and stigma scales need clearer description of scoring, thresholds, and validation in this population. | We thank the reviewer for this comment. The manuscript has been revised to clarify the scoring, thresholds, and validation of the scales used. Stigma was measured using the Abridged HIV Stigma Scale, a validated multi-item instrument assessing perceived HIV-related stigma. Responses were scored and summed to generate a total stigma score, which was then categorized into low and high stigma based on predefined thresholds. Food insecurity was assessed using the Household Food Insecurity Access Scale (HFIAS). Additional details on scoring and categorization have been added to the Methods section to improve clarity. | Page 5 (Line 141 – 144) |
| **Interpretation appears overstated** |  |  |
| Some adjusted ORs have extremely wide confidence intervals (e.g., 1.1–35.4; 1.2–38.1), suggesting imprecise estimates due to small cell counts. | We thank the reviewer for this observation. We acknowledge that some adjusted odds ratios had wide confidence intervals, likely due to small cell counts or limited sample sizes for certain categories. This limitation has been added to the manuscript to highlight the imprecision of these estimates. | 14 ( Line 276 – 277) |
| The discussion occasionally implies causation, which is inappropriate for a cross-sectional design. | We thank the reviewer for this observation. We have revised the discussion to avoid implying causation, emphasizing that the findings reflect associations rather than causal relationships due to the cross-sectional study design. | Page 15 - 16 |
| **Writing, structure, and clarity** |  |  |
| Several grammatical errors, missing words, and formatting issues require revision. | We appreciate the reviewer’s comment. The manuscript has been carefully proofread and revised to correct grammatical errors, fill in missing words, and standardize formatting throughout to ensure clarity and readability. | Whole manuscript |
| The background section mixes national statistics with global information in a way that feels unfocused. | We thank the reviewer for this observation. The Background section has been reorganized to clearly separate global context from national statistics, providing a more focused and logical presentation of the study rationale. | 1 and |
| **Minor Concerns** |  |  |
| Some references are outdated or inconsistent in formatting. | We thank the reviewer for this comment. All references have been reviewed and updated to include the most recent sources where applicable, and formatting has been standardized according to the journal’s guidelines. | 20-22 |
| Tables are lengthy and may benefit from consolidation or improved formatting. | We appreciate the reviewer’s suggestion. The tables have been reviewed and reformatted for clarity. | Table 1 – 4 |
| Ethical section appears duplicated and should be edited for clarity. | We thank the reviewer for noting this. The Ethics section has been revised to remove duplication and edited for clarity, presenting the ethical approval, informed consent, and confidentiality procedures in a concise and coherent manner. | Page 6 (Line 162 – 168) |
